# Role of healthcare practitioners in rotavirus disease awareness and vaccination – insights from a survey among caregivers

**DOI:** 10.1080/21645515.2019.1632685

**Published:** 2019-07-16

**Authors:** Bernd Benninghoff, Priya Pereira, Volker Vetter

**Affiliations:** Global Medical Affairs, GSK, Wavre, Belgium

**Keywords:** Awareness, parents, rotavirus, gastroenteritis, survey, vaccination

## Abstract

An online survey was designed to assess awareness and understanding of Rotavirus (RV) gastroenteritis (RVGE), and knowledge and attitudes towards RV vaccination in Germany, Poland, Turkey, Indonesia, the Philippines and Thailand. Survey participants (n = 1500) comprised parents, expectant parents and guardians of children ≤5 years of age who have sole or joint responsibility for health and well-being decisions relating to their child, who were recruited from an online panel and provided their consent for study participation. Participants from most countries had a high level of awareness of RV infections (mean: 82%) and of those aware of RV, a mean of 61% participants were aware that RV was the most common cause of GE, however the majority (mean: 59%) were unaware that nearly every child would be infected with RVGE by the age of 5 years. Healthcare professional (HCP) recommendation was identified as the key driver for participants seeking vaccination (48%–75% of participants stated this reason, with results differing by country) followed by availability of RV vaccine in the national immunization program. Despite a high level of awareness of RVGE among participants, fostering knowledge regarding the difficulty of RVGE prevention, the risk of RV contraction and the associated serious consequences like dehydration is imperative to improve RV vaccination uptake. HCPs, being the primary influence on participants’ decision on vaccination, are best suited to bridge existing knowledge gaps and recommend parents to vaccinate their children against RVGE.

## Introduction

**Figure:**
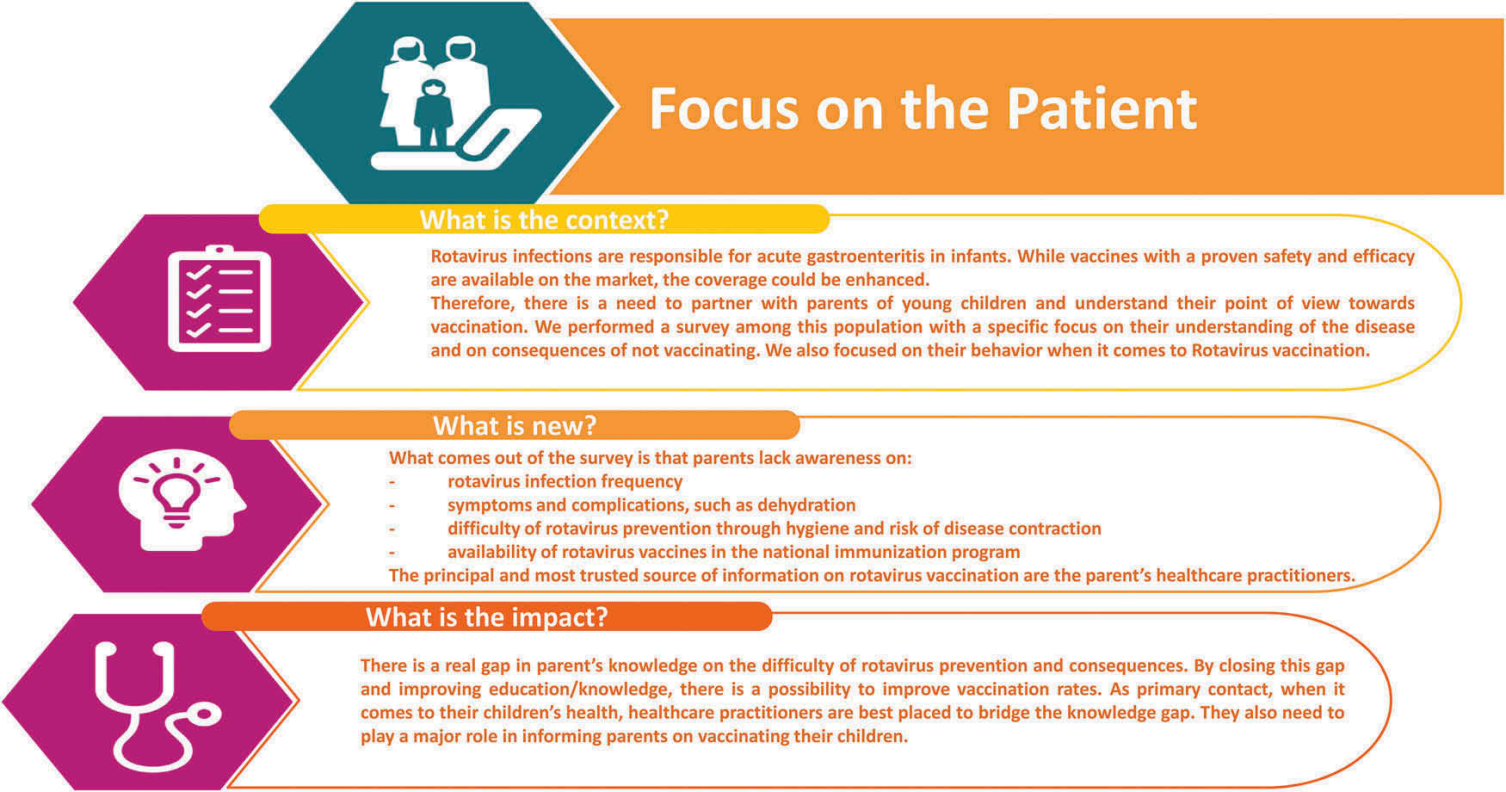

Rotavirus (RV) infection is the primary cause of viral acute gastroenteritis (GE) in infants and children <5 years old.^1,2^ It is estimated that by the age of 5 years, nearly every child will have had at least one episode of rotavirus gastroenteritis (RVGE).^3^ Although maintaining good hygiene like handwashing, sanitation along with exclusive breastfeeding for 6 months, vitamin A supplementation and safe drinking water are essential to prevent the spread of infections, RV vaccination has proven to be the most effective approach to protect children against RVGE disease.^1,4^ The clinical characteristics of RVGE can be severe and include enteric symptoms such as fever, vomiting, diarrhea and dehydration. Fundamentally, the clinical severity and diverse outcomes of RVGE in infants are known to exert substantial emotional and social impact (direct and indirect) on their families.^1–3^ RVGE affects young children at a crucial time of physical and emotional development with consequences on cognitive development and it has also been suggested that diarrhea in childhood is linked to adult chronic diseases such as cardiovascular diseases, diabetes, obesity, and hypertension.^4–6^ As RVGE is characterized by a sudden onset it can disrupt daily routines of the family which can require unexpected changes, and can consequently affect the physical, emotional and social well-being of the child and its family.^1,7–9^ One study reported that changes in parents’ daily routines were more often documented if the child with RVGE had a fever or insufficient fluid intake.^7^ In other studies, parents reported experiencing moderate or severe parental distress, worry and anxiety, as well as intense feelings of exhaustion, helplessness and despair due to RVGE; notably this distress was not necessarily easily comprehended by healthcare professionals (HCPs).^1,8–10^ A study conducted in Italian parents showed that during RVGE hospitalization, mean parental stress scores (out of 10, with 10 as highest stress) ranged from 6.6 to 8.4. The highest scores were documented for the presence of malaise (8.42) followed by the occurrence of vomiting/diarrhea (8.07) and child dehydration (7.18). The overall stress for the family was graded as ‘high’ by 67.2% of parents.^8^ In another study conducted in the United States, parents frequently reported feelings of frustration and helplessness when they could not help their sick child, or reported being frightened by the severity of the illness and becoming fatigued by caring for their child.^11^ In both high- and middle-income settings, the lack of awareness about RVGE could contribute to the stress, anxiety and fear that parents experience when they have a child with RV infection.^1,7,8,12^ The social and emotional impact of RVGE in infants results in considerable loss of family income due to days off work, and societal costs due to reduced productivity or absenteeism.^9,11,13^ Prevention of this disease through prophylactic vaccination could vastly improve the daily lives of children and parents.^14^

**Figure 1.**
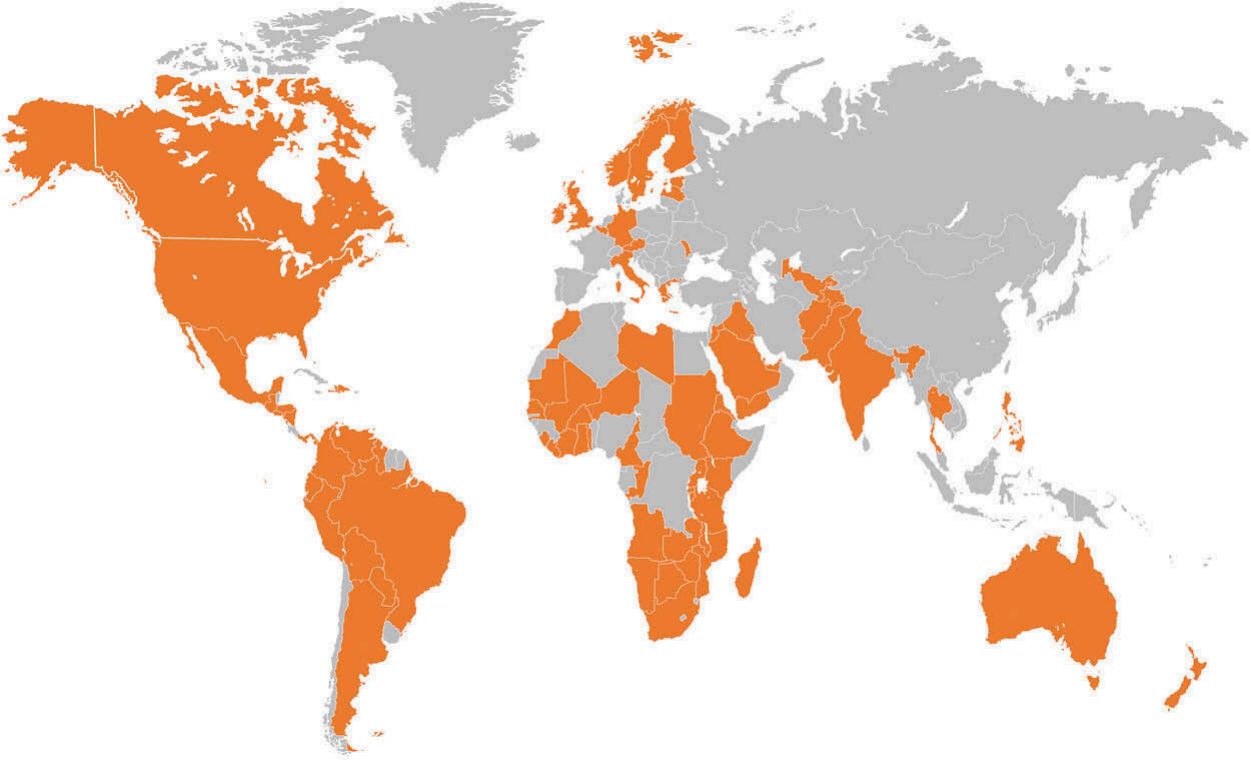
RV vaccination in NIPs worldwide. NIP, national immunization program; RV, rotavirus. Source: International Vaccine Access Center^23^

The World Health Organization (WHO) and national health authorities recommend the implementation of RV vaccinations in all national immunization programs (NIPs), regardless of a country’s level of development and particularly for those countries where diarrheal disease is a major health problem.^15–17^ Prior to the introduction of RV vaccines, nearly 453,000 deaths among children under the age of five years due to RVGE and 35%-40% of hospital admissions were reported globally in 2008.^18^ Following the availability and use of RV vaccination, this figure dropped to between 128,500 and 146,000 deaths by 2016.^19,20^ Thus post-vaccination, the mortality rates due to RVGE substantially declined primarily in high-income settings and the hospitalization rates were positively impacted (50–80%).^21,22^ As of 2018, RV vaccine has been introduced in the NIP of 97 countries (Figure 1) (with 90 countries having introduced a universal mass vaccination program [UMV]),^23,24^ meaning that less than one-third of the world’s birth cohort has access to RV vaccines.^25,26^ Although deaths due to RVGE have declined, there remains a significant direct and indirect disease burden due to the large numbers of RVGE cases, hospitalizations and associated complications.^16,19,27,28^ Specifically, RVGE-related hospitalizations are known to overstretch and clutter emergency departments and primary healthcare facilities given the concurrent seasonality of RV and other common childhood infections in most areas of the developed world.^16,27^ This results in additional economic burden in terms of direct healthcare costs associated with medical practitioner visits, hospitalizations, and strain on the healthcare system.^13,28^

Despite the availability of safe and efficacious vaccines, RV vaccination remains underutilized in infants, which is in contrast with observed vaccination coverage rates with most primary childhood vaccines which have remained stable and high.^22,25,29^ The reasons for underutilization are similar in both high- and low-income settings (Text box 1)^30,31^ A common misconception among parents is that improved hygiene and cleanliness would prevent cases of RVGE. However, the fact that pre-vaccine RVGE rates are universally similar indicates that hygiene practices are not sufficient to reduce transmission of RV.^10^ In high- and middle-income settings, the fact that morbidity due to RVGE is perceived as unlikely can influence the willingness of HCPs to recommend RV vaccination and parents to vaccinate their children against RVGE.^8,32,33^ The use of in-depth interviews with individuals involved in childhood vaccination decision-making such as parents and guardians of children, may help elicit a greater understanding and awareness of important issues around the RV disease and the use of RV vaccine.

**Table 1.** Overview of participants included in the survey.

Participants	Germany (n = 250)	Poland (n = 250)	Turkey (n = 250)	Indonesia (n = 250)	Thailand (n = 250)	Philippines (n = 250)	Total (n = 1500)
Parents of infants and children ≤5 years	226	228	237	237	245	236	1,409
Expectant parents (first child)	24	22	13	13	5	14	91
**Gender**							
Male	68	87	135	120	79	76	565
Female	182	162	115	130	171	174	934
Prefer not to say	-	1	-	-	-	-	1
**Age**							
0–18 years	-	-	-	1	1	-	2
19–25 years	25	66	27	27	39	32	216
26–35 years	133	127	157	153	139	153	862
36–45 years	84	49	63	65	67	57	385
46–55 years	7	5	3	2	3	4	24
≥56 years	-	-	-	-	-	-	-
Prefer not to say	1	3	-	2	1	4	11
**Working situation**							
Working full time (≥30 hours/week)	131	156	209	188	185	160	1029
Working part time (8–29 hours/week)	57	18	8	34	29	41	187
Not working	46	59	31	23	26	40	225
At school/university/in education	6	11	1	4	5	2	29
Prefer not to say	10	6	1	1	5	7	30
**Education level**							
Degree or degree equivalent and above	63	110	193	191	154	172	883
Higher education below degree level	66	106	26	53	24	61	336
A level or equivalent	100	25	28	6	12	6	177
Trade apprenticeships/GCSE level or below	19	5	3	-	58	4	89
No qualifications	-	-	-	-	2	4	6
Prefer not to say	2	4	-	-	-	3	9

GCSE, General Certificate of Secondary Education; N, number of respondents.

**Table 2.** Disease and vaccine awareness.

Awareness of RV	% mean value	% range (by country)
**RV versus other diseases**	82%	62%-88%
**About RVGE (based on those aware of RV)**		
– RV is the most common cause of gastroenteritis	61%	53%-68%
– RV cannot be eliminated through proper sanitation/hygiene	56%	50%-66%
– Almost every child will have had RV by age 5	41%	36%-48%
– In severe cases of dehydration due to RV, children may need to be admitted to hospital	76%	63%-87%
**Perceived seriousness of RVGE (very/extremely serious)**	82%	75%-88%
**Top mentions for awareness of RV symptoms (based on those aware of RV)**		
– Watery diarrhoea/stools	75%	68%-81%
– Vomiting	69%	63%-87%
– Fever	63%	58%-74%
– Abdominal pain	59%	48%-75%
– Dehydration	57%	41%-75%
– Loss of appetite	52%	42%-66%
RV vaccine awareness*(based on those aware of RV)	41%	28%-57%
Believe that vaccines are available via NIP**	52%	37%-64%
**Believe that vaccines are available via private market**	61%	38%-76%
**Child has received RV vaccine via NIP**	26%	24%-57%
**Raises the subject of childhood vaccination**		
– Doctor	69%	55%-81%
– Myself	21%	10%-34%
– Nurse	10%	2%-19%

*Versus other vaccines. **Data excludes Indonesia – vaccine available in the NIP was not presented to respondents as an option. NIP, national immunization program; RV, rotavirus; RVGE, rotavirus gastroenteritis.

**Text box 1.** Common reasons for RV vaccine underutilization among parents and / or HCPs.

Given misconceptions around RV Attitudes of providers Questions around the need for the vaccine Questions around the effectiveness of the vaccine Concerns about safety of the vaccine

Source: Kempe et al,^30^ Veldwijk et al^31^

In this multi-country online survey conducted across countries in Europe and Asia, we aimed to acquire insights from participants (parents and guardians of children) about their awareness and understanding of RVGE, available interventions and mediums used to gain disease information to help facilitate the development of effective communication strategies to improve RVGE awareness among parents and families of young infants. The survey also aimed to understand factors influencing participants’ perceptions and attitudes towards RVGE, explore attitudes towards the need and value of a RV vaccine and identify the barriers to vaccination and the impact of such barriers on participants’ willingness to vaccinate their children.

## Materials and methods

### Study design and participants

A quantitative 10-minute online survey was conducted between December 2017 and January 2018 in Germany, Poland, Turkey, Indonesia, the Philippines and Thailand. The countries were selected based on three criteria: (i) the type of RV vaccination program present in the country to capture any similarities or differences in attitudes or knowledge in different settings. For example, there is a 100% reimbursed UMV program for RV vaccination in Germany (strong recommendation) whereas in Thailand and the Philippines a phased or regional vaccination program exists (moderate recommendation) and in Indonesia, Turkey and Poland, there has been no decision or introduction of RV UMV, (ii) willingness to participate in the survey, and (iii) countries that had a low uptake of RV vaccination which implied that these countries could greatly benefit from the results of the survey.

Selection of study participants was based on willingness to participate to the study, and therefore not randomized. All participants were recruited via an online consumer panel, wherein they had opted into being contacted for research purposes. Eligible participants were parents and guardians of infants or children ≤5 years of age (and a small sub-sample of expectant parents) who were solely or jointly responsible for the family’s health and well-being decisions, were open to vaccinating their children and who chose to participate in the research on attitudes towards disease and vaccination.

The survey was composed of 26 questions related to family settings, participant knowledge of RV disease, its symptoms, vaccine availability, and preferred source of information. To ensure quality, the survey was launched in one market first (Germany) with a full data check conducted to ensure the reliability of the survey prior to launch in all countries. Participants were adequately informed about the aims of the study and those who consented to enter the survey were provided with a structured questionnaire that consisted of multiple choices and open questions. The survey questionnaire was made available in English as well as the local language. An honorarium at fair market value was offered to respondents in all countries for taking part in this survey. The survey was conducted in accordance with local applicable guidelines.

### Analysis

The survey questionnaire aimed to collect data with respect to knowledge of RV disease, including awareness of the disease, its symptoms, risks, and prevention strategies; and knowledge of vaccines, including awareness of available vaccines, their importance, and drivers or barriers to vaccination. Data collected from the questionnaire were analyzed overall across the six countries as well as at country-level. No differentiation was made based on the economic or educational level of respondents.

Descriptive statistics were presented using the number and percentage of responses for each question. Total mean values were calculated by dividing the sum of all the values by the number of participants and weighted equally across all countries. Data in this publication is shown at an overall level, including respondents from all participant groups. Data for expectant parents only were not analyzed separately due to the low number of respondents that were included in the survey.

## Results

### Respondents

A total of 1,500 participants were recruited from six countries, with 250 participants from each country: Germany, Poland, Turkey, Indonesia, the Philippines, and Thailand. The majority (94%) of participants were parents and guardians of infants and children ≤5 years old and the remaining (6%) of participants were expectant parents. Most participants were females (62%) of ages between 26 and 35 years (57%) who were working full time i.e. 30 hours or more per week (69%) and were educated to a degree level or above (59%) (Table 1).

### Disease awareness

Survey results on RVGE disease awareness are provided in Table 2. In most countries, the vast majority of participants were aware of RVGE disease, with a mean awareness level of 82% (range: 62%-88%). However, the participants’ awareness level of RV being the most common cause of GE was reported to be lower (mean: 61%; range: 53%-68%) in all countries. In addition, only 56% of participants in each country (range: 50%-66%) were aware that RV cannot be eliminated by handwashing and maintenance of hygiene.

Most of the participants were unaware that almost every child would have had a RV infection by the age of 5 years, with a mean awareness of only 41% (range 36%-48%) demonstrating a lack of knowledge of the high-risk disease transmission characteristics. The lowest level of awareness was seen among participants from Indonesia, with only 36% previously aware of this information. The highest level of awareness was observed in Thailand though 52% of participants still cited this information as ‘new’.

The mean awareness level of the link between RVGE and dehydration was only 57% (range: 41%-75%). Less than half the participants from Indonesia and Thailand were aware of dehydration as a symptom of RVGE. Awareness was the highest in Poland, where 75% of participants were aware of the link between RVGE and dehydration. Most participants were aware of the serious consequences of severe dehydration in a child (mean: 76%; range 63–87% aware that dehydration can result in hospital admission); though knowledge on the link between RV and dehydration was lower.

### Vaccine awareness

Survey results on RV vaccine awareness are provided in Table 2. Less than half of the participants in all countries were aware of the existence of a RV vaccine (mean: 41%), with the lowest level being reported in Indonesia (28%), where the vaccine is available privately, and the highest level in Germany (57%) where the vaccine is available through a NIP. Participants’ awareness about some other childhood vaccines (chickenpox, flu, measles, meningitis [meningitis and encephalitis – Thailand], mumps, rubella [German measles], hepatitis [Thailand only]) was observed to be higher compared to the RV vaccine.

**Figure 2.**
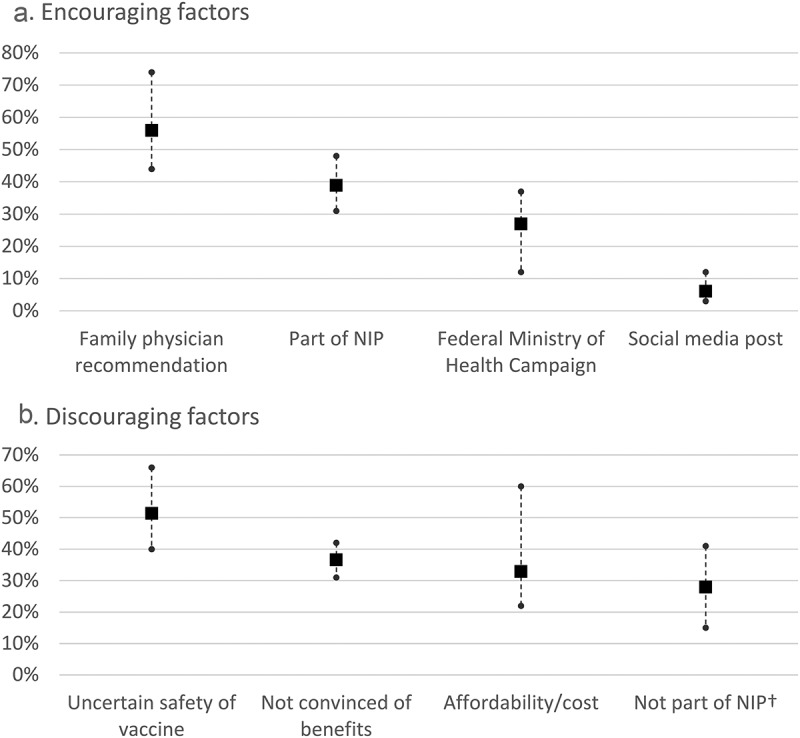
Top three factors encouraging and discouraging participants to seek RV vaccination. MoH, ministry of health; NIP, national immunization program; RV, rotavirus. Solid squares represent overall participant mean values, solid circles represent min and max countries values.

In all countries, family physician or pediatrician recommendation was the greatest driver of participants seeking vaccination for their children. A mean of 56% (range 44–74%) selected family physician recommendation as one of the top three most influential factors followed by the availability of the vaccine in the NIP (mean 39%) and campaigns by Federal Ministry of Health (mean of 27%) (Figure 2). In all countries uncertainty about the safety of the vaccine was considered as the biggest discouraging factor for vaccination (mean: 52%; range: 40–66% selected as one of the top three factors). Furthermore, “not being convinced of the benefits of vaccination” was cited as one of the top three key discouraging factor in most countries (mean: 37%; range: 31–42%), followed by affordability/cost reasons (mean 33%; range 22–60) and the unavailability of the vaccine via the NIP (mean: 28%; range: 15%-41%; Germany, Turkey, Thailand and the Philippines only). In all countries, participants indicated that the doctor was the main initiator of vaccination-related discussions (mean: 69%; range: 55%-81%). In Poland, 34% of participants highlighted that they initiated conversations with their doctors. Similarly, in Indonesia, 33% of participants reported that they usually raise the discussion about vaccination, although participants’ awareness about the disease is the lowest of all countries. It is possible that the participants aware of the disease and vaccine are a little more receptive to online tools and social media for information and advice on vaccination, explaining why they may raise the conversation with HCP more frequently. This could also conceivably be due to the pilot vaccination scheme in the region.

**Figure 3.**
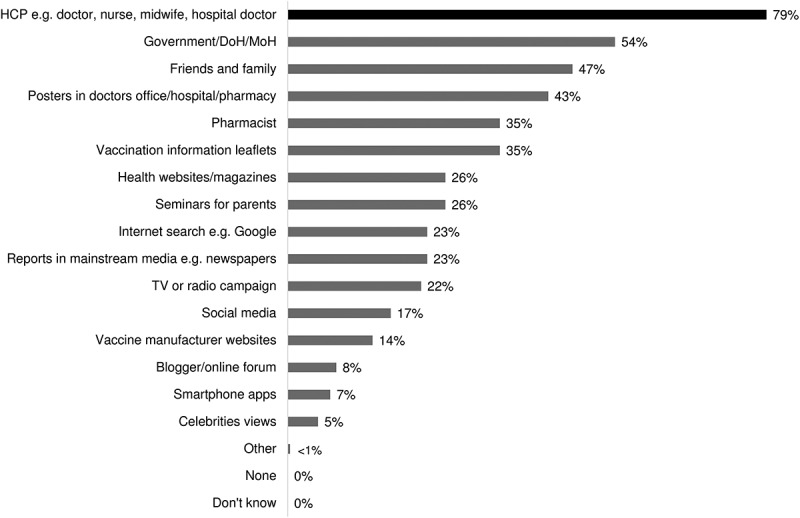
Most likely channel for information/advice on vaccines. HCP, healthcare practitioner; DoH, department of health, MoH, ministry of health; TV, television.

In most countries, face-to-face conversations with HCPs were regarded as the preferred source of information on vaccination. Almost 80% of participants reported that HCPs would be one of the top five sources they would turn to for information and advice related to vaccination. The second most likely source of information on vaccines reported was the government or the Ministry of Health (with a mean of 54% selecting this as one of the top five sources they would turn to). Family and friends, posters in doctor’s offices/hospitals/pharmacies, pharmacists and vaccination information leaflets were also cited (with a mean of 47%, 43%, 35% and 35% of participants selecting each respectively as one of their top five sources of information). The use of seminars, online tools such as social media was also preferred by a small proportion of participants (with a mean of 17%) (Figure 3).

## Discussion

This survey utilizing online questionnaire-based interviews, aimed to provide insights about the level of participants’ knowledge and perceptions on RVGE disease and the need for prevention through vaccination, as well as to seek information on the sources used by participants to access disease- and vaccination-related information.

The awareness of RVGE among participants was generally high across most of the countries investigated except for participants from Indonesia. Strikingly, most participants (mean: 59%) were not aware that by age 5, most children would have had an RV infection. Despite a high level of knowledge about RVGE among participants in this survey, the difficulty of disease prevention despite proper hygiene and sanitation was new information to a significant proportion of participants. Importantly, almost half of the surveyed participants (mean: 44%) were unaware that RV cannot be eliminated by handwashing and the daily use of disinfectants. These findings are urgent to address because it could undermine the development of effective RV control strategies. Further to this, results of an Internet survey conducted by the Canadian Institute of Child Health in 2007 in Canada showed that only 48% of the 822 surveyed Canadian mothers (with at least one child under the age of 3 years) had heard of RV.^34^ The contrast with our results (62%-88%) could be explained by several factors. Knowledge about the disease may have globally spread with the first introduction of the vaccine in 2006. Indeed, Patel et al have observed that awareness about the disease in the US at the same period was low, although this was not quantified.^35^ Additionally, the use of an online survey in our case might have contributed to selecting participants with a higher education and economic level. Considering these different observations, further research to elucidate the awareness of RVGE disease and identifying the potential determinants of RVGE awareness are needed to help close this knowledge gap.

A cross-sectional study conducted in Sweden showed that parents did not consider RVGE as a serious illness.^36^ While there was a prevailing high level of awareness among participants that dehydration led to hospitalization, our study highlighted a high level of unawareness towards dehydration being an important symptom for RVGE disease (mean: 57% aware). The link between RV gastroenteritis and dehydration was the highest (75%) in Poland; although no government reimbursed universal RV vaccination has been adopted in this country. This could possibly be related to a higher parental level of education, which has been linked to health literacy and choices about vaccination.^37^ Although the participants in our study sample may have a higher education level or be more affluent than their country average due to the selection method used, this may be particularly the case for Poland, where levels of education are already high.^38,39^

Additionally, the specific role of breastfeeding in the prevention of RVGE has not been well established; however, it is generally considered that it reduces the severity of the disease and can help prevent against dehydration that results in hospitalization.^40^ However, several studies now confirm that although breastfeeding is important in prevention, it does not provide protection against severe dehydration caused by RVGE.^41–43^ A previous study conducted in Italy reported that parental distress due to RVGE-related hospitalization (of which dehydration is known to be the main driver) was significant (93.6% reported high or medium stress). The highest scores for stress were attributed to child dehydration among others.^8^ Specifically educating parents about a stronger link between RV and dehydration could consolidate the parents’ awareness of the RVGE disease seriousness. Increasing awareness about the frequency and potentially serious consequences of RVGE is of paramount importance and could be the key drivers in increasing RV vaccination uptake.

This survey further revealed that there was a low awareness of the existence of a RV vaccine (mean: 41%, range: 28%-57%) and that confusion exists on how to have access to the vaccine, with many participants believing it existed in the NIP (63% Germany, 64% Thailand, 57% Philippines, 37% Turkey, 37% Poland) and some believing their child had already received the vaccine through this channel regardless of whether it is or isn’t an option in their country (57% Germany, 42% Thailand, 24% Turkey, 33% Philippines). Low awareness about the existence of the vaccine highlights the need to educate the parents on the efficacy and safety of vaccination, especially in markets where parents may need to pay out-of-pocket for the vaccine. Improving awareness about the ease of administration of this vaccine is also needed since after the oral poliovirus vaccine, RV vaccine is the only orally administered vaccine given to children under the age of 6 months. Similar surveys conducted in European countries have also shown that out-of-pocket expenses or vaccine cost, vaccine effectiveness, duration of protection and frequency of severe adverse effects were factors influencing parents when deciding about vaccination against RV for their children.^31,44^ A survey of parents from Canada revealed that the main reasons why parents who had positive intentions yet did not have their child vaccinated were that they believed the child was receiving enough vaccines, that RV vaccine was not useful and RV vaccine was not included in the free public vaccination program.^32^ Providing clarity on how to have access to the vaccine and its easy administration, the early successes of RV vaccination program introduction in 97 NIPs as of 2018 and its cost could potentially contribute to increasing the RV vaccination rate.^23,24^

This survey further revealed that participants believed HCPs (e.g. family doctor, nurse, midwife, hospital doctor) to be one of their top five sources of information when seeking vaccination for their children (mean: 79%). The findings of this study were consistent with those of the previously published study conducted in Sweden, which confirms the critical role of HCPs in providing information on vaccination.^33^ In a qualitative survey conducted in the US, most parents were unaware of the health burden of RV disease, however, most of them expressed a likelihood of adopting vaccination for their children, if their physicians recommended it.^35^ In another survey study conducted in Canada, it was reported that parents who opted to vaccinate their children were mainly informed by HCPs, which highlighted the positive influence of HCPs’ recommendations on vaccination behaviour.^32^ Various other studies have also supported the HCPs’ role in providing health education and raising awareness about the importance of vaccinating children to improve vaccination rates.^8,45^ In Indonesia, a study of HCPs showed that they did not consider diarrhea to be an important problem in the country, but they did acknowledge that it could be serious if not properly treated. While many HCPs had some level of knowledge about RV, not all knew that a vaccine was available. There were also mixed feelings towards the need for the vaccine, some HCPs felt that the vaccine was not ranked as a priority as it was not listed on the national program.^10^ This conception from the HCPs in Indonesia may explain the low awareness of participants about RV symptoms and vaccine availability, as this study shows that HCPs are their most important source of information about the vaccine. Improving the acceptance of HCPs towards RV vaccine is important in low- and middle-income countries since their attitude will impact parents’ decisions on vaccinating their children. In India, a survey revealed that factors such as the relationship with the pediatrician and vaccination-related decisions taken by people in the immediate social network were important drivers for decisions about vaccination against RV.^46^ Our survey showed that other channels including the government/department of health/ministry of health websites, family and friends, disease/vaccination awareness posters in doctor’s offices, hospitals, or pharmacies, pharmacists and vaccination information leaflets were also influential. Social media could also play a role, especially in Indonesia and Thailand, where approximately a quarter of participants rank it in their top five most likely channels to refer to (a mean of 24% in Indonesia and 27% in Thailand). An advertisement campaign to parents in conjunction with the ministry of health websites, social media and leaflets with a message to parents stating, ‘ask your doctor about RV vaccination’ could help raise the vaccine conversation with HCPs.

However, the internet, forums and social networking tools have allowed anti-vaccination advocacy groups to have a broader reach than ever before.^47^ Considering the growing anti-vaccine sentiment in some countries and the fact that misinformation about vaccination is often disseminated through social media,^48^ health authorities should realize the proportion of parents obtaining their health information through social media. Messages from anti-vaccination groups through social media should be examined and countered.^48^ Hence it is essential that trusted sources of vaccine information validated by reputed public health agencies such as WHO,^49^ European center for disease prevention and control (ECDC),^47^ centers for disease control and prevention (CDC)^50^ among others are available and shared with the public to improve vaccination advocacy.

The use of an online survey to elicit a greater understanding of participants’ awareness is a key strength of our work. However, we acknowledge that there are limitations associated with this study. Prior to engaging in the survey, participants were informed that the survey was about ‘people’s attitudes towards vaccination and specific diseases’, hence there may have been a selection bias for participants with positive perceptions towards vaccination. In addition, the generalization of the findings may be limited due to a potential selection bias as the research was conducted using an online panel of consumers. Results from this survey may not be nationally representative as the population samples in this survey from each of the six countries comprised of well-resourced, connected individuals who chose to take part in the survey; this could have led to an overestimation of awareness of participants about RVGE disease. Therefore, the results of this study may not be generalizable to poorer populations in low-income countries, and further studies about awareness should be performed, particularly in low-income countries, including those eligible for vaccine introduction support from Gavi. It must also be noted that the total level mean scores are based on an equal weighting for each country and were not weighted to reflect the population size of each country. Lack of an interviewer or pre-test to ensure participants’ ability to understand the questions might have also influenced the responses and findings of the study. Furthermore, some participants responded to the survey based on recalling their experience up to 5 years prior and hence were likely to be subjected to recall bias.

In summary, understanding of the RV disease and acceptance of vaccination among individuals involved in decisions on vaccination for their children must be achieved through a proper communication channel. Educating parents about the high risk of RV contraction and potentially serious consequences of the disease, especially due to dehydration, could possibly drive them to seek a solution and consult relevant stakeholders about vaccination. The role of HCPs is very significant in the vaccine decision-making process; therefore, attention should be brought to propose adequate training to HCPs to improve counseling and communication about the value of RV vaccination. Parents usually turn to HCPs for advice; in response HCPs should facilitate discussions surrounding RV vaccination so that parents can make a well-informed decision about vaccinating their children to prevent RVGE disease.
